# Microglial migration and interactions with dendrimer nanoparticles are altered in the presence of neuroinflammation

**DOI:** 10.1186/s12974-016-0529-3

**Published:** 2016-03-22

**Authors:** Fan Zhang, Elizabeth Nance, Yossef Alnasser, Rangaramanujam Kannan, Sujatha Kannan

**Affiliations:** Center for Nanomedicine, Johns Hopkins University School of Medicine, Baltimore, MD 21231 USA; Department of Materials Science and Engineering, Johns Hopkins University, Baltimore, MD 21218 USA; Anesthesiology and Critical Care Medicine, Johns Hopkins University School of Medicine, Baltimore, MD 21287 USA; Department of Ophthalmology, Wilmer Eye Institute, Johns Hopkins University School of Medicine, Baltimore, MD 21231 USA; Hugo Moser Research Center, Kennedy Krieger Institute, Baltimore, MD 21205 USA; Present address: Department of Chemical Engineering, University of Washington, Seattle, WA 98195 USA; Pediatrics, Johns Hopkins University School of Medicine, Baltimore, MD 21287 USA

**Keywords:** Microglia, Inflammation, Morphology, Cell migration, Dendrimer

## Abstract

**Background:**

Microglial cells have been implicated in neuroinflammation-mediated injury in the brain, including neurodevelopmental disorders such as cerebral palsy (CP) and autism. Pro-inflammatory activation of microglial cells results in the impairment of their neuroprotective functions, leading to an exaggerated, ongoing immune dysregulation that can persist long after the initial insult. We have previously shown that dendrimer-mediated delivery of an anti-inflammatory agent can attenuate inflammation in a rabbit model of maternal inflammation-induced CP and significantly improve the motor phenotype, due to the ability of the dendrimer to selectively localize in activated microglia.

**Methods:**

To elucidate the interactions between dendrimers and microglia, we created an organotypic whole-hemisphere brain slice culture model from newborn rabbits with and without exposure to inflammation in utero. We then used this model to analyze the dynamics of microglial migration and their interactions with dendrimers in the presence of neuroinflammation.

**Results:**

Microglial cells in animals with CP had an amoeboid morphology and impaired cell migration, demonstrated by decreased migration distance and velocity when compared to cells in healthy, age-matched controls. However, this decreased migration was associated with a greater, more rapid dendrimer uptake compared to microglial cells from healthy controls.

**Conclusions:**

This study demonstrates that maternal intrauterine inflammation is associated with impaired microglial function and movement in the newborn brain. This microglial impairment may play a role in the development of ongoing brain injury and CP in the offspring. Increased uptake of dendrimers by the “impaired” microglia can be exploited to deliver drugs specifically to these cells and modulate their functions. Host tissue and target cell characteristics are important aspects to be considered in the design and evaluation of targeted dendrimer-based nanotherapeutics for improved and sustained efficacy. This ex vivo model also provides a rapid screening tool for evaluation of the effects of various therapies on microglial function.

**Electronic supplementary material:**

The online version of this article (doi:10.1186/s12974-016-0529-3) contains supplementary material, which is available to authorized users.

## Background

Microglial cells are the primary resident immune cells in the central nervous system (CNS). Their migration dynamics are associated with their functions in the CNS [1–3]. Under physiological conditions, microglia are ramified cells with highly motile processes to help survey and maintain the brain microenvironment around them [4]. In response to any acute brain injury or damage, these surveying microglial cells can rapidly transform into an activated state and migrate to the injury site [5–8]. However, in neurodevelopmental disorders such as cerebral palsy (CP), where activated microglial cells have been implicated, the migration dynamics of microglial cells are not well-understood [9]. Previous studies using primary microglial cell cultures have demonstrated that lipopolysaccharide (LPS) suppresses microglial migration and process extension, while IL4 and TGF-β promote microglial migration and branching [9–11]. Nevertheless, microglial migration dynamics in a more representative biological environment, such as the brain parenchyma, especially in the presence of pathology, have not been adequately explored.

Microglial cells have the ability to phagocytose stressed or dying neurons and express phagocytic receptors on their surface [12]. In neuroinflammatory and neurodegenerative disorders, pro-inflammatory microglia become neurotoxic by secreting reactive oxygen species and cytokines as a response to various environmental stimuli, causing injury to neurons [13–15, 38]. We have previously demonstrated that systemic administration of a poly(amidoamine) (PAMAM) dendrimer (~4 nm) results in its selective accumulation in activated microglia in the brain of newborn rabbits with CP but not in healthy, age-matched control rabbits. We have also shown that newborn kits with CP had evidence of ongoing inflammation and oxidative injury in the brain even on day 5 of life (8 days after the insult). When *N*-acetyl cysteine (NAC), a broad anti-oxidant and anti-inflammatory agent with poor brain penetration, was conjugated to dendrimers and administered systemically on day 1 of life (3 days after the insult) to rabbit kits with CP, a dramatic improvement in motor function and attenuation of neuroinflammation was noted by day 5, and was significantly more effective than free drug at a 10-fold higher dose [16]. However, since the dendrimers do not cross the intact blood brain barrier (BBB) in healthy rabbits, it is unclear what role the “activated” nature of pro-inflammatory microglial cells play in the dendrimer uptake, as the BBB impairment in this rabbit model of CP would allow a greater amount of dendrimer exposure within the brain parenchyma to the pro-inflammatory microglial cells.

To address these questions, and further understand the dynamic functions of microglial cells in this in vivo rabbit model of CP, we created an ex vivo organotypic whole-hemisphere brain slice culture model, with preservation of the microglia pathology in an in vivo condition. Using this platform, we evaluated microglial migration and interactions with dendrimers in brain slices obtained from newborn rabbits with CP, and compared to that of healthy control newborn rabbits. We found that inflammation led to impairment in the surveillance function of microglial cells, as demonstrated by hindered migration of microglial cells in the brain of newborn kits with CP compared to healthy control kits. Inflammation also influenced the mechanism of microglia-dendrimer interactions, with enhanced and more rapid dendrimer uptake by the microglial cells in the brain slices from kits with CP. A better understanding of the dynamics of microglial migration in the presence of inflammation and their interactions with dendrimer nano-devices will provide valuable information for evaluating and designing targeted drug delivery systems that can be used to modulate microglial function and interactions.

## Methods

### Reagents

Cyanine5 NHS ester-labeled hydroxyl terminated Generation 4.0 Poly(amidoamine) (PAMAM) dendrimer (D-Cy5 or referred to as dendrimer in this paper) was synthesized using a previously established protocol [17]; Tomato lectin—DyLight 594 (Vector Lab, USA); Goat anti-Iba1 (Abcam, USA); Donkey anti-goat-Alexa flour 488 (Invitrogen, USA); and 4′,6-diamidino-2-phenylindole (DAPI) (Invitrogen, USA) were purchased and used for the studies.

### Animals

All animal procedures were in accordance with the Animal Care and Use Committee guidelines at Johns Hopkins University and the United States Department of Agriculture (reference number: RB14M324), as described previously [18, 19]. Timed pregnant New Zealand white rabbits were obtained from Robinson Services Inc. (Winston-Salem, NC). Briefly, pregnant rabbits in the endotoxin/CP group underwent laparotomy at gestational day 28 (term pregnancy is 31 days) and were injected with 1 mL of saline containing *Escherichia coli* endotoxin (~6000 EU) (serotype O127: B8, Sigma Aldrich) along the length of the uterus. At this dose, the newborn kits have been shown to have uniform pro-inflammatory microglial activation in the periventricular region (PVR), increased expression of TNF-α, and display a phenotype of CP with predominantly hindlimb hypertonia [16, 19]. The healthy control group included pregnant rabbits that had no surgery or intervention. All pregnant dams were induced on the evening of gestational day 30 (G30) to control timing of delivery, and kits were used for the experiments on postnatal day 1, corresponding to G31.

### Organotypic whole-hemisphere brain slice preparation

Organotypic whole-hemisphere brain slices were prepared based on modifications to previously published protocols [5, 20, 21]. Rabbit brain slices (350-μm thick) were prepared from neonatal rabbits with CP or from age-matched healthy controls. To prepare the brain slices, neonatal rabbits were decapitated under aseptic conditions after euthanasia. The brain was removed, dissected into two hemispheres, and sectioned immediately into 350-μm thick whole-hemisphere brain slices using a Mcllwain tissue chopper (TED PELLA, Inc., USA). For each hemisphere, six consecutive slices at the level of the bregma were carefully separated in the dissection medium (3.2 g glucose/500 ml HBSS, 1 % of penicillin), while maintaining the structures of whole-hemisphere brain slices intact. The lateral ventricle was clearly visualized in all the slices. The separated brain slices were transferred onto 30-mm diameter, sterile, porous (0.4 μm) transparent and low-protein-binding membrane inserts (Millicell-CM, Millipore) in six-well tissue culture plates. Each well was prefilled with 1 mL of culture medium, prepared from 200 mL MEM, 100 mL HBSS RED, 100 mL Horse Serum, 4 mL Glutamax, and 1 % penicillin. The slices were maintained overnight at 37 °C in a humidified atmosphere with 5 % CO_2_ before confocal imaging. For the slice viability studies, new culture medium was replaced every 2 days.

### Evaluation of the viability of brain slices under culture

The whole-hemisphere brain slice viability was evaluated using lactate dehydrogenase (LDH) assay (Cayman, USA), which measures the LDH released into the culture medium from degenerating cells in brain slices [22]. Culture supernatants from three different CP slices were collected at various time points up to 10 days of incubation and replaced with fresh medium at each time point (time points evaluated were 6 h, 19 h, 27 h, day 2, day 3, day 5, day 6, day 7, day 10) and frozen at −80 °C. The dilution of LDH in the supernatant was taken into account in the calculation. The percentage of LDH released in each whole-hemisphere brain slice was quantified by measuring the fluorescence intensity, subtracting the background in negative control (culture medium at 0 h of slice culture) and normalized by the intensity of positive control (culture medium collected from brain slices treated with Triton-X 100 for 10 days).

### Time-lapse imaging of microglial migration

Time-lapse imaging of microglial cell migration in brain slices was carried out using a LSM 710 inverted fluorescence confocal microscope (Zeiss, USA). In preparation for imaging, whole-hemisphere brain slices from neonatal rabbits with CP and their age-matched controls were carefully removed from the insert after overnight incubation and simultaneously transferred to a lysine-coated glass-bottom culture dish (MakTek Corp, USA). Control and CP brain slices were stained and imaged in pairs simultaneously to maintain the same conditions between groups. To stain the microglial cells, brain slices were incubated with 10 μL of Tomato Lectin 594 added to 1 mL of culture medium for 45 min and then washed three times with the medium to remove unreacted tomato lectin. To fix the slice onto the glass-bottom culture dish, 30–40 μL of matrigel were applied around the brain slices and then incubated at 37 °C for 15 min to allow curing. Culture medium without phenol red was then added into the glass-bottom culture dish such that it just covered the brain slices. Time-lapse imaging was carried out in the environmental chamber under 37 °C and 5 % CO_2_ to allow the brain slices to maintain their normal physiology. Prior to imaging, the environmental chamber was pre-equilibrated for 30 min. To decrease the possibility of any laser-induced injury to the cells and photobleaching of the fluorescence labeled dendrimers, a low laser power index of 0.5 was used. Image quality was maintained by using a comparatively larger pinhole size (90 μm) and higher gain number to compensate for the decrease of laser power. A 20× tilescan with a Z-stack of 2–5 μm interval in the vertical direction was used to image a tissue dimension of 1 mm × 1 mm in area, 50–80 μm in thickness at the PVR. An average of 40–50 microglial cells were imaged per slice. The movie was recorded at a temporal resolution of 15 min for 5.5–6 h. Maximum intensity projection was applied for the final image process.

### Evaluation of dendrimer uptake by microglial cells

To evaluate the dendrimer uptake by microglial cells, freshly prepared whole-hemisphere brain slices from rabbits with CP and age-matched healthy controls were incubated with dendrimer tagged with Cy5 (D-Cy5) (5 ng in 10 μL of sterile Dulbecco’s phosphate-buffered saline (DPBS) solution) 4 h after slices were sectioned. The temporal profile for the evaluation of dendrimer uptake during the whole experiment window is shown in Additional file 1: Figure S1. The D-Cy5 solution was topically pipetted on the tissue along the medial border of the lateral ventricle (Additional file 1: Figure S1). This region is typically where microglia are seen in high density in newborn rabbits [18, 23]. The slices were then incubated at 37 °C in the presence of 5 % CO_2_ to allow dendrimer to diffuse though the brain tissue and interact with microglia. Slices were collected after 1, 4, and 12 h of treatment. At the end of the treatment, the slices were washed with cold DPBS solution (4 °C) to inhibit active mechanisms of cell uptake and to remove excess dendrimers that were not taken up intracellularly. Brain slices were then carefully transferred to 4 % formalin solution and 20 % sucrose solution for fixation. Slices were incubated overnight with Goat Anti-Iba1 for microglia, followed by donkey anti-goat Alexa flour 488 as secondary and DAPI for nuclear staining. The stained brain slices were imaged with a 20× lens with 3 × 3 tilescan, and Z-stack with a thickness of 100 μm. The parameters for Cy5 channel were kept consistent throughout all images to allow for comparison.

### Image analysis

#### MATLAB analysis of microglia movement

The recorded cell migration was analyzed using MATLAB software. For each group (CP vs. healthy), three slices, each obtained from different animals, were analyzed. Microglia movement rates were obtained by transforming the coordinates of microglia centroids into time-averaged mean square displacement (<MSD>), calculated as < MSD(*τ*) > = [*x*(*t* + *τ*) − *x*(*t*)]^2^ + [*y*(*t* + *τ*) − *y*(*t*)]^2^, where *x* and *y* represent the cell coordinates at a given time and *τ* is the time scale or time lag. The acquired <MSD> was analyzed using the diffusion function in a 2D scale < MSD (*τ*)> =4*Dτ*^*α*^, where *D* is the diffusion coefficient, which in our case, is related to the microglial cell migration. *τ* is the time lag and *α* is the dynamic exponent. When *α* = 1, the diffusion or cell movement tends to be Brownian motion. When 0 < *α* < 1, the diffusion or cell movement is considered to be hindered. When *α* > 1, the diffusion or cell movement is considered to be active transport which is governed by other factors, such as chemokine or cytokine sensing.

### Analysis of microglial migration velocity and persistent distance

The analysis of microglial migration velocity and persistent distance was based on the time-lapse videos recorded by LSM710 confocal microscopy. The speed of microglial migration was determined by tracking microglial cell migration path using image analysis software in Metamorph. Information, such as coordinates and distance at each time point, was recorded. Based on this information, the migration speed and persistent distance were calculated by averaging the migration velocity at each migration step. The persistent distance is defined as the distance (≥10 μm) traveled by a microglial cell before it makes a significant change in direction (absolute angle between previous direction and new direction <70°) [24].

### Imaris analysis of microglial cell surface to volume ratio (S/V) and dendrimer co-localization

To evaluate the change in morphology of the microglia in brain slices from CP kits when compared to those from age-matched healthy control newborns, 3D representations of Anti-Iba1 stained microglia in fixed whole-hemisphere brain slices were acquired using confocal microscopy with ×40 magnification, with 3 × 3 tile scans, extending 10 μm in the Z direction with 1-μm Z-stacks. To analyze the image, Imaris software was applied and microglial surface to volume (S/V) ratio was measured. The function “surfaces” was used, and the microglia from different slices (*n* = 3 slices/group from three different kits each, for the CP and control groups) were analyzed for S/V ratio at the beginning of incubation and 24 h post incubation [10].

To study the dendrimer uptake by microglial cells, we used the “spot” function to detect microglial cells and dendrimer accumulation. For the detection of dendrimer accumulation, a threshold was set, where only dendrimers with signal beyond this threshold were considered detectable. The “co-localization” function was used to determine whether cell uptake was present. In detail, the estimated diameter chosen was 7, 10, and 10 μm, respectively, and the threshold was automatically set for “Quality” analysis to 5000, 4000, and 7000, respectively. The function “co-localize spots” was used and a 10-μm distance threshold was chosen.

### Statistical analysis

Statistical analysis of data was carried out by using student’s *t* test, and one-way ANOVA. Differences were considered statistically significant at *p* < 0.05. For MSD analysis, mixed model ANOVA was used.

## Results and discussion

### Results

#### Whole-hemisphere brain slice viability and microglial pathology is maintained throughout the experiment window

**Fig. 1:**
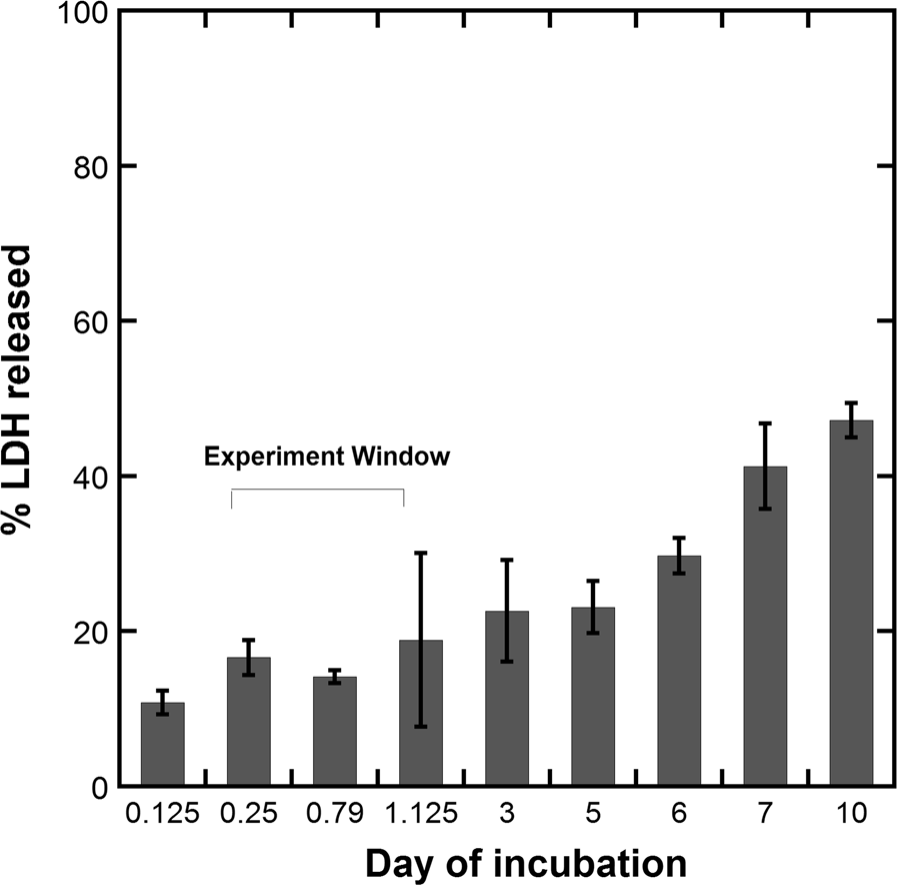
LDH release by neonatal rabbit brain slices as an indicator of tissue viability during incubation. Percentage of LDH released in neonatal rabbit brain slices during 10 days of incubation period. The released LDH concentration is an indicator of the extent of cell death in the whole brain slices. All confocal based experiments were conducted within the experiment window. For each time points, three brain slices from CP kits were used. For statistical analysis, one-way ANOVA was conducted (refer to Additional file 1: Table S1)

Assessment of LDH activity from the medium has been widely used to quantify cell death in primary cell cultures. Similarly, in tissue slice culture, the measurement of LDH efflux into the culture medium has been found to be highly correlated with the results of cell death from cell counting [25]. To determine the cellular viability of the slices, the LDH levels in the supernatant were measured. All microglial studies were conducted between ~0.2 and 1 days (4–24 h) post sectioning, during which time no significant increase in LDH level was observed. (Figure 1; Additional file 1: Table S1), indicating that there was no increase in cell death over time during the observation period. This implies that the tissue stability was maintained during this period. We found that the brain slices maintained good viability during the time frame when these experiments were conducted, without an increase in LDH release.

**Fig. 2:**
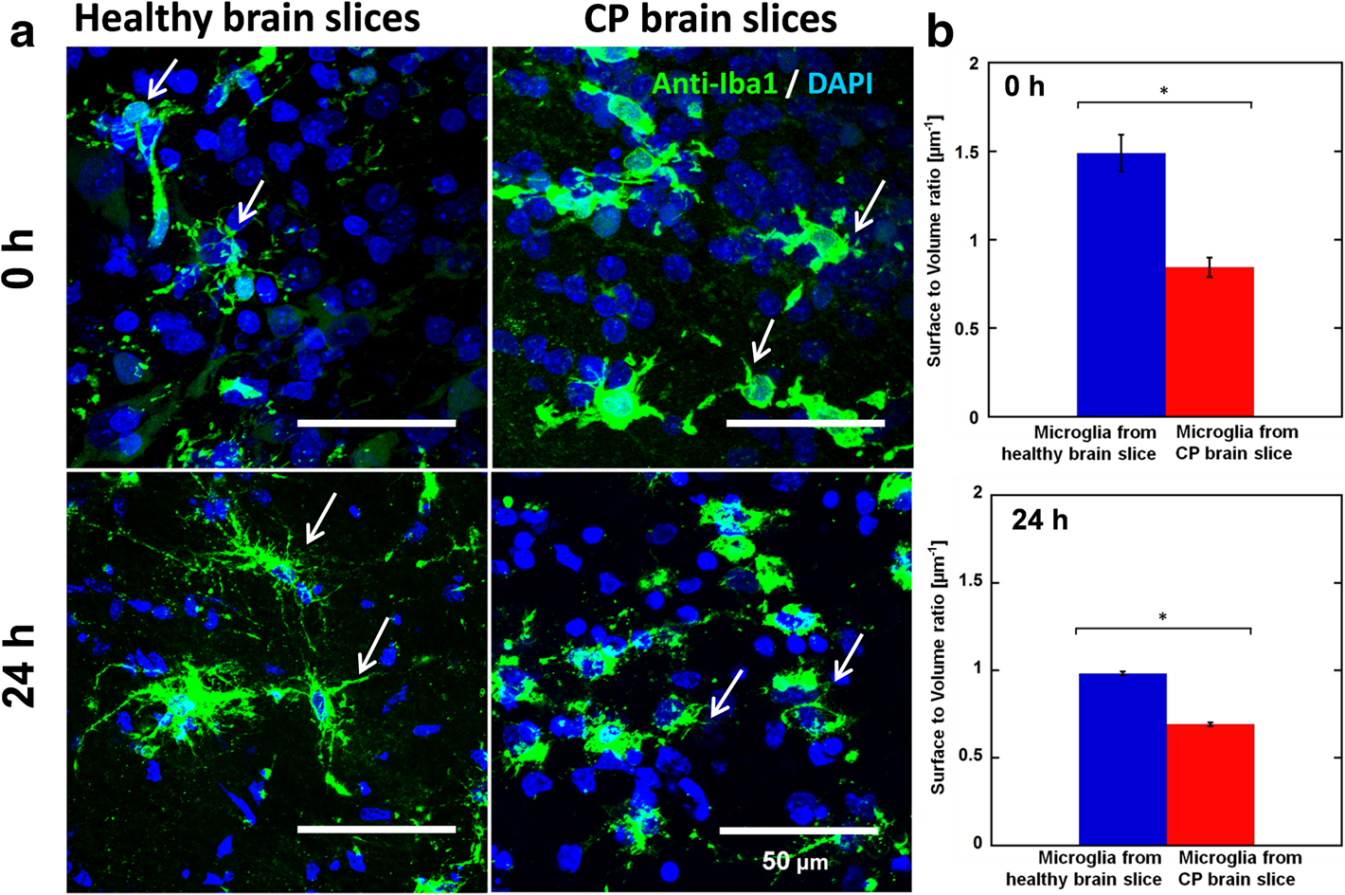
Microglial cells morphology is different in CP brain slices and healthy brain slices within the experiment window. **a** Representative morphology of microglia cells from cerebral palsy (CP) brain slices and healthy brain slices at 0 and 24 h after incubation; **b** Quantitative study of microglia cell morphology from CP brain slices and healthy brain slices 0 and 24 h after incubation; the morphology was characterized by cell surface area to cell volume ratio. *p < 0.05 (data shown as mean ± SEM). *Blue*: DAPI,* green*: anti-Iba1 (antibody labeling microglia). *Scale bar* in the figure, 50 μm. For each group, images were acquired from at least three different brain slices

To validate whether the acute study (experiment window within 24 h) allowed the preservation of in vivo pathology of microglial cells, we evaluated the differences in microglial morphology between CP and healthy control kits over time in brain slices by quantifying microglial surface area to volume (S/V) ratio (Fig. 2). Microglial cells in the brain slices from healthy control kits demonstrated several extended processes with small cell bodies, while microglial cells in the brain slices from CP kits had enlarged cell bodies with several short, thickened processes (identified with arrow in Fig. 2). These morphological variations lead to differences in the microglial cell S/V ratio, with S/V ratio close to 1.5 μm^−1^ in the healthy controls and S/V ratio less than 1 μm^−1^ in the CP kits. At 24 h, the end of our experiment window, a statistically significant difference in S/V ratio between microglia in the healthy brain slices and CP brain slices could still be observed. This difference was noted despite a generalized decrease in the S/V ratio of microglia in both healthy and CP brain slices over time. This decrease over time from baseline is probably related to a normal response of the microglia to the shear force generated during tissue section. In our in vivo model of CP, we observed a similar difference in morphology with decreased branches and larger cell bodies in the microglia in kits with CP compared to healthy control kits [16].

Specifically, the viability of neonatal brain slice culture platform was evaluated by measuring the LDH released in the supernatant during 10 days of incubation time (Fig. 1). In general, cells in the brain slices showed increasing degeneration rate with longer incubation time, with ~10 % of total LDH released at the beginning of incubation and ~50 % at the end of incubation (10 days). Although there was ~10–20 % LDH release caused by shear force induced cell damage during the slicing of the brain prior to incubation, cells in the brain slices quickly equilibrated with the ex vivo media culture environment, as indicated by the stable level of LDH for the first 24 h of incubation. LDH levels remained stable for 3–5 days of incubation time after slicing, indicating no increase in cell death and maintenance of tissue viability during this time period which provided an experiment window long enough for the studies of cell mobility and dendrimer uptake.

#### Microglial migration is different in brain slices from CP animals compared to brain slices from healthy control animals

To investigate the influence of inflammation on microglial migration, we used the mean square displacement (<MSD>) of individual microglia to quantify the migration in the brain slices. We found a higher fraction of “active migration” for microglial cells in the brain slices from healthy control animal slices and a higher fraction of “restrictive migration” for microglial cells in CP, indicating impaired migration of microglia in the CP rabbit brain.

**Fig. 3:**
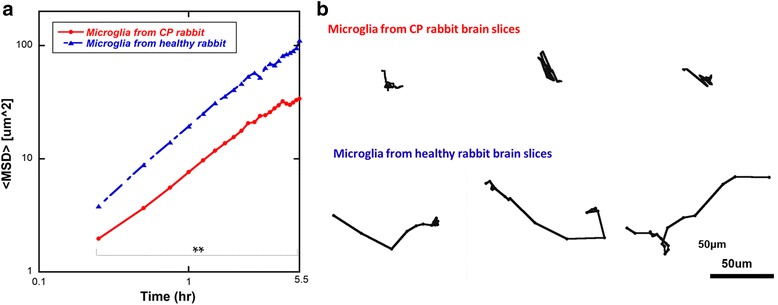
Microglial migration is impaired in brain slices from CP animals vs. healthy animals. **a** Microglial cells from cerebral palsy rabbit brain slices (*red*) had lower <MSD> and diffusion coefficient compared to microglial cells from healthy rabbit brain slices (*blue*). The <MSD> was plotted in a log scale as a function of time. Mixed model ANOVA analysis showed that the migration of microglia from healthy brain is different from that from the CP brain. **b** Representative trajectories of microglia from cerebral palsy rabbit brain slices and healthy rabbit brain slices are shown. **p < 0.01

**Fig. 4:**
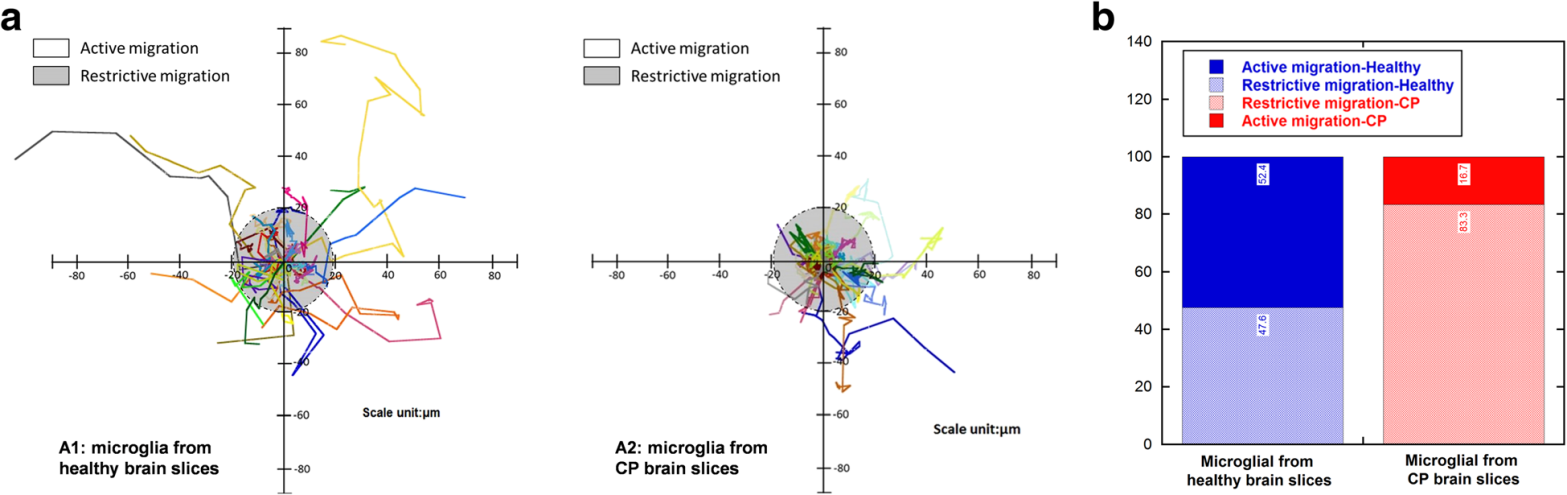
Microglia trajectories showed higher percentage of active migrating microglial cells in healthy brain slices. **a** Overlay of individual microglial cells trajectories, plotted after aligning their starting positions. Cells were tracked over a 6-h period. Units are in micrometers. Restrictive migration was defined as microglial cells migrating within 20 μm from their starting point (*shaded area*); active migration was defined as migration of more than 20 μm from their starting point. *A1*: 42 microglial cells from healthy brain slices; *A2*: 48 microglial from CP brain slices. **b** The quantitative study demonstrated higher “active migrating” microglia population in healthy rabbit brain slices. In the brain slices from healthy rabbits, more than half the microglia cells (52.4 %) showed active migration pattern, while 83.3 % of microglia from CP rabbit brain slices showed restrictive migration pattern

Throughout the imaging time span of 5.5 h, the average <MSD> for the microglia was greater in the control brain slices compared to CP slices. The increased migration was reflected in the higher “diffusion coefficient,” which is the intercept of the log plot of the <MSD> vs. time graph (Fig. 3a and Additional file 1: Figure S2, Additional file 2). This means that in the healthy brain slices, microglia traveled greater distances on average than microglia in the CP brain slices, within the same period of time. This could be due to a combination of microglial cytoskeletal changes, retraction of microglial processes, and changes in the extracellular matrix resulting in the decreased microglial movement. The microglial trajectories were individually plotted and migration distance from the starting point was measured. Microglia in the control brain slices had a much greater migration distance compared to those in the slices from CP rabbit kits (Fig. 3b). To quantify this difference, we classified all microglial migration trajectories as “active transport” or “restrictive transport.” Specifically, the microglial migration trajectories in both groups of slices were re-plotted by aligning the starting point of each trajectory at the same origin and by defining the cell migration with displacement greater than 20 μm as active transport while displacements less than or equal to 20 μm were considered as restrictive transport (Fig. 4a). Over one-half the microglia in the healthy slices (52 %) had an active migration pattern with a migration distance more than 20 μm from their starting point; while only 17 % of microglial cells in the CP brain slices demonstrated active migration, 83 % of microglial cells in the CP brain slices demonstrating restrictive migration (Fig. 4b).

**Fig. 5:**
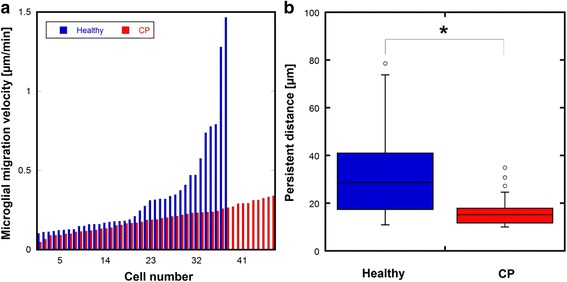
Microglial cells from CP brains showed lower migration velocity and less persistent distance. **a** Microglial migration velocity distribution for healthy and CP brain slices. Microglia from healthy brain slices showed higher average migration velocity during the observation period, especially at high migration velocity range, than the microglia from CP brain slices. **b** Persistent distance of microglial migration trajectory is greater in the healthy rabbit brain slices. Persistent migration was defined as the length (>10 μm) traveled by a microglial cell before it makes a significant change in direction (absolute angle between previous direction and new direction <70°) *p < 0.05 For microglia from healthy brain slice, population median 30.3 μm, 20 % percentile, 16.6 μm, 80 % percentile, 44.4 μm; for microglia from CP brain slice, population median 15.22 μm, 20 % percentile, 11.6 μm, 80 % percentile, 17.9 μm

To investigate the difference in microglial migration between the healthy and CP slices, we used migration velocity and persistent distance to evaluate migration patterns (Fig. 5). In general, microglial cells had a wide distribution of migration velocities. Microglial cells from healthy brain slices had migration velocities ranging from ~0.1 to 1.5 μm/min. This range was wider, and the velocities were higher, than those of microglial cells from CP brain slices, which had migration velocities ranging from ~0.05 to ~0.3 μm/min. This resulted in a higher average microglial migration velocity in healthy brain slices (~0.33 ± 0.31 μm/min; mean ± SEM) than in CP brain slices (~0.19 ± 0.08 μm/min). Persistent distance is a parameter used to measure the interactions of the cell with its matrix regulated by adhesion proteins [24]. We adopted this parameter and used it as an indication of impediments encountered by microglia during migration. The lower persistent distance demonstrated by the microglial cells from CP brain slices compared to healthy slices (population median 15.2 vs. 30.3 μm) may suggest there is a greater hindrance to migration for impaired microglia.

#### Dendrimer uptake is affected by microglial pathology

We have previously reported that intravenous administration of dendrimers resulted in increased accumulation in activated microglia in newborn rabbits with CP, but not in healthy controls [16, 17]. A similar differential uptake was found between the microglia in healthy control brain slices and in CP brain slices.

**Fig. 6:**
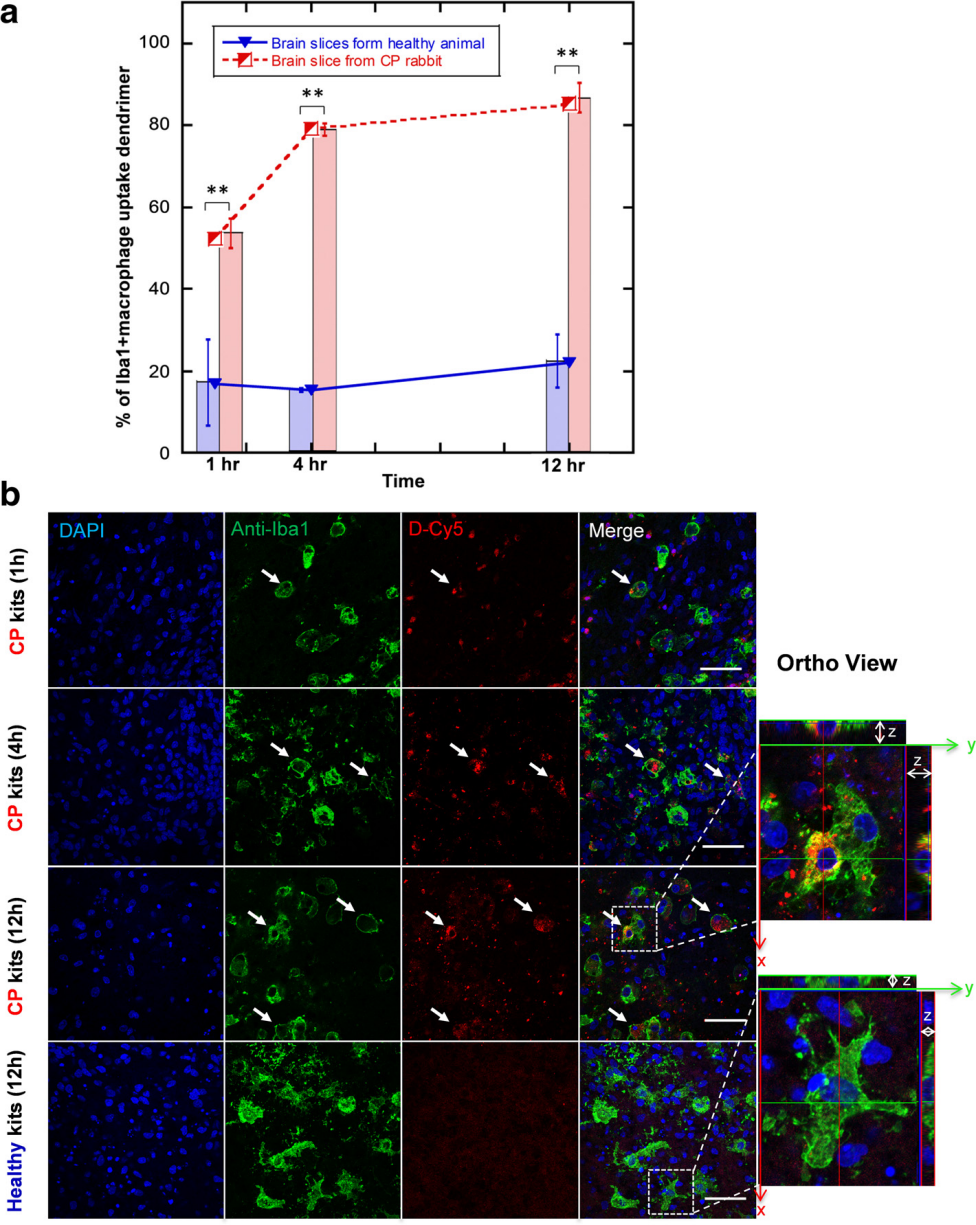
Quantitative study of microglia-dendrimer interactions in CP and healthy brain slices. **a** Dendrimer uptake by microglial cells, expressed as percent of Iba1+ cells that co-localize with D-Cy5 as a function of time (mean ± SEM). *Red*: D-Cy5 uptake in microglia from CP brain slices; *blue*: D-Cy5 uptake in microglia from healthy brain slices. *N* = 3 slices/group. **p < 0.01. **b** Confocal image of dendrimer treated brain slices (CP, taken under ×63 magnification) at three different time points after D-Cy5 treatment (1, 4, 12 h) and healthy brain slices after 12 h of D-Cy5 treatment. Microglial cells in CP brain slices had greater dendrimer uptake (indicated by *white arrow*) than in healthy slices. The orthographic projection clearly indicated the dendrimer uptake by activated microglial cells in the brain slice from CP kit, while the dendrimer was barely seen to localize in microglial cells in the brain slice from healthy kit. *Blue*: DAPI, *green*: anti-Iba1 (antibody labeling microglia), *red*: D-Cy5. *Scale bar*, 50 μm

In the brain slices from healthy animals, less than 20 % of microglial cells had detectable dendrimer uptake after 1 h of dendrimer treatment (Fig. 6a). The uptake remained at the same level, without obvious increase in uptake even after 12 h of dendrimer treatment, indicating limited interactions between dendrimer and microglial cells in the healthy brain slices. This can be observed in Fig. 6b, where the dendrimer signal in healthy slices was barely observed. In brain slices from CP kits, dendrimer uptake by microglial cells occurred more rapidly and to a greater extent, with about half of microglial cells containing dendrimer after 1 h treatment. Microglial uptake of D-Cy5 in the brain slices from CP kits increased 1.6-fold after 4 h of dendrimer treatment, with ~80 % of total microglial cells containing dendrimer (Fig. 6a). No obvious increase in the number of microglial cells containing dendrimer was observed beyond this time point, suggesting that dendrimer uptake by microglial cells peaks around 4 h in vivo without substantial increase in uptake beyond this time point. This was also depicted in Fig. 6b and Additional file 1: Figure S3, where substantial co-localization of D-Cy5 in microglia can be seen by 4 h of treatment in the brain slices from CP kits, with relatively less cellular co-localization observed in the healthy brain slices even at 1, 4, and 12 h (Fig. 6b, and Additional file 1: Figure S3).

### Discussion

In this study, we show that maternal intrauterine endotoxin exposure results in a morphological change of the microglia with retraction of the processes, restrictive microglial migration, and a more rapid and enhanced dendrimer uptake in the newborn rabbit brain with CP when compared to age-matched healthy controls. These observations provide insights to why the dendrimer-drug conjugates are therapeutically effective, since the dendrimers diffuse more rapidly in the brain (and are able to “find” the less mobile activated microglia), and can take advantage of the increased ability of microglia increased ability to engulf/uptake dendrimers.

In vitro and in vivo models are commonly used to study the morphology, behaviors, and functions of microglia. In vitro platforms, including primary cell cultures, allow the extraction of high purity microglial cells [26]; however, in vitro methods involve destruction of the primary architecture and biological environment of the CNS. The physiology or pathology is well maintained in in vivo platforms, but it is experimentally difficult to visualize and monitor the biological process in specified anatomical locations, especially for regions deep within the brain [27]. On the other hand, the ex vivo organotypic whole-hemisphere brain slice platform maintains many aspects of in vivo characteristics, such as the 3D structure of the brain, making it possible for microglial cells to interact with the biological environment around them [5, 28, 29]. Using organotypic whole-hemisphere brain slices as a platform in this study also allows us to access and precisely monitor the cells at a specific anatomical location [30], i.e., the PVR which is the primary area involved in CP. Since the goal of this study was to evaluate the interactions of the dendrimer with microglial cells, while avoiding the biological barriers such as the BBB and clearance by other organs, the organotypic whole-hemisphere brain slices were an appropriate ex vivo platform. Here, we transformed an in vivo animal model of CP into an ex vivo brain slice model of CP. Our results suggest that these differences in microglial morphology between the two groups, as observed in our in vivo model, were also maintained in our organotypic slices [19].

The study of microglial migration is associated with the understanding of microglial function in the brain and how they might be affected differently under acute injury and inflammation. In the healthy brain, microglia serve a housekeeping function by continually surveilling the brain microenvironment, pruning neurons, and clearing accumulated metabolic products or deteriorated tissue components in parenchyma [4, 31]. In vivo studies in the adult mouse brain have revealed that, to perform these functions, microglial cells are highly dynamic, where their processes continuously undergo cycles of de novo formation and withdrawal (on a time scale of a few minutes) without obvious movement of their cell somata [4]. However, under conditions of acute insult, such as laser-induced neuronal injury or blade induced lesion, microglial cells quickly react to these insults and transform into a motile mode with increased migration [4, 32–34]. This finding was demonstrated in ex vivo studies in neonatal rats/mice organotypic brain slices [5, 35]. The shear force generated during the slicing process induces mechanical stimulation. Microglia under normal physiologic condition react to this stimulus by quickly transitioning into a motile mode within a time period of a few hours and translocating their cell somata. This enables direct physical contact with many cells within a short period of time and supports a tissue surveillance function [5]. This was also demonstrated by microglial cells in the brain slices from healthy control animals in our study.

However, in the presence of inflammation, microglial cells behave differently. Inflammation induced by LPS can polarize microglial cells into a pro-inflammatory phenotype [36, 37]. Studies in primary microglial cell cultures have found that exposure to LPS led to decreased microglial migration. Although the mechanisms are not fully understood, LPS has been shown to impair microglial migration by downregulation of the P2Y(12) receptor, which promotes microglial migration in response to ATP/ADP release [10, 11]. Similarly, in our slices obtained from newborn rabbits with CP, microglial migration decreased, as shown by lower <MSD> and reduced migration speed, with a higher population of microglia characterized by restrictive migration when compared to the microglia in the healthy brain. This restricted and decreased migration would indicate the impairment in the normal surveillance function of the microglia. It is possible that this impairment in surveillance function of pro-inflammatory microglia may play a role in mediating ongoing, chronic inflammation in neuroinflammatory/neurodegenerative disorders [13, 38]. Persistence of cell migration, a measure to evaluate the ability to migrate in the same direction without turning, has been used in studying cell migration, including for cancer cells [39]. The persistent distance of the cell migration trajectory may be regulated by multiple mechanisms, including microtubules, adhesion proteins, and chemotactic signals [24, 40–42]. In this study, it was found that microglia demonstrate reduced persistent distance in the presence of inflammation. This may be explained in part by increased microglial adhesion to the ECM component laminin, due to the upregulation of the membrane protein integrin on microglia when exposed to pro-inflammatory cytokines [43, 44]. Integrin expression is known to regulate the adhesion and migration of cells in the CNS [43].

In our maternal inflammation-induced rabbit model of CP, we have previously reported an increase in the uptake of systemically administered hydroxyl terminated PAMAM dendrimer (dendrimer) by “activated” microglia in the newborn brain of kits with CP but not in the healthy neonatal rabbit brain [16]. In the in vivo model, the impairment of the BBB in the CP animals may lead to greater exposure of the microglia to the dendrimer in the brain in these animals. However, in the ex vivo model, microglia in the healthy brain slices and the CP animal derived brain slices are exposed to the dendrimer to the same extent, due to direct contact of the dendrimer to the slice. The substantially greater and more rapid uptake of dendrimers by microglia in the tissue of CP rabbits when compared to healthy controls may indicate upregulation of the cellular uptake mechanisms by the microglia in CP kits. This is in spite of some levels of “activation” in both groups due to the shear forces induced by slicing the brain tissue. Microglial cells possess a full range of endocytic mechanisms, including receptor-mediated endocytosis, macropinocytosis, and phagocytosis [45]. Upregulation of any or all of these processes in pro-inflammatory microglial cells in the CP brain [12] could be responsible for the enhanced dendrimer uptake and intrinsic targeting of the dendrimers that can be exploited for targeting therapeutics specifically to activated microglia [16, 46–49].

## Conclusions

We used an ex vivo brain slice platform that maintained the physiology of surveying microglial cells and pathology of pro-inflammatory microglial cells from an in vivo model. Our results suggest there is a significant decrease in the normal migration and surveillance functions of microglia in the presence of neuroinflammation in newborn rabbits with CP. However, these microglia were able to uptake dendrimer more rapidly and to a greater extent than normal healthy control microglia. Further studies to define the endocytotic processes that are upregulated in activated pro-inflammatory microglia will help to better understand the mechanisms by which the dendrimer is taken up by activated microglia. This will also help in designing better nanoparticle platforms to target activated microglia.

## Additional files

Additional file 1:
**Figure S1**: the demonstration of experiment schematic; **Figure S2**: the comparison of the mean square displacement of microglial cells from three sets of brain slices (CP v.s. healthy); **Figure S3**: Confocal image of 1 h and 4 h dendrimer treated brain slices from healthy kits. **Table S1** – One-way ANOVA analysis for Figure 1. (DOCX 15914 kb) Additional file 2: The real-time video of microglia migration in the brain slices from CP and healthy kits. Scale bar 100 μm. For each brain slice, the vedio was recorded during 6 h time period, with a temporal resolution of 15 min. (MP4 12959 kb)
