# Supplementary material for: Microglial migration and interactions with dendrimer nanoparticles are altered in the presence of neuroinflammation
Source: J Neuroinflammation. 2016 Mar 22;13:65. doi: 10.1186/s12974-016-0529-3 (PMC4802843; doi:10.1186/s12974-016-0529-3)
Supplement: Additional file 1: — Figure S1: the demonstration of experiment schematic; Figure S2: the comparison of the mean square displacement of microglial cells from three sets of brain slices (CP v.s. healthy); Figure S3: Confocal image of 1 h and 4 h dendrimer treated brain slices from healthy kits. Table S1 – One-way ANOVA analysis for Figure 1. (DOCX 15914 kb) [file 12974_2016_529_MOESM1_ESM.docx]

# Additional files

### Figure S1 – Experiment Schematic


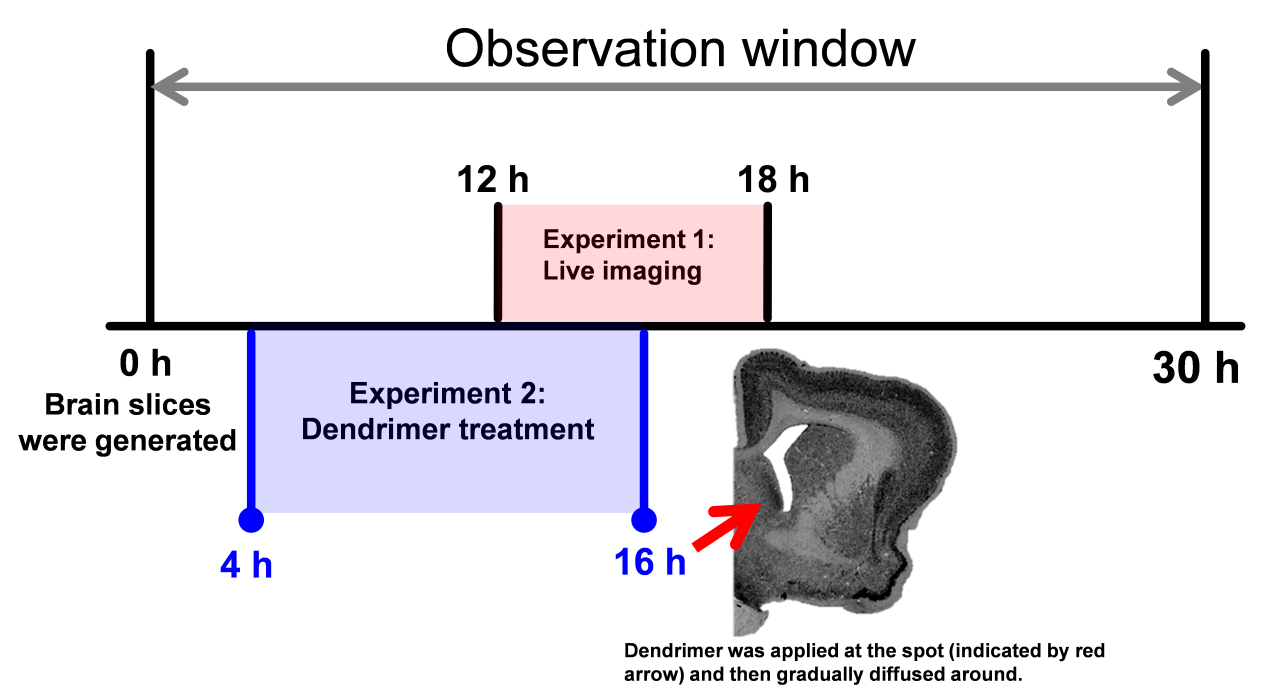


This schematic describes the timeline for the migration and dendrimer uptake experiments.

### Figure S2 – Comparison of the mean square displacement <MSD> of microglial cells from CP (Red) and Healthy (blue) slices during live-imaging in 3 different brain slices


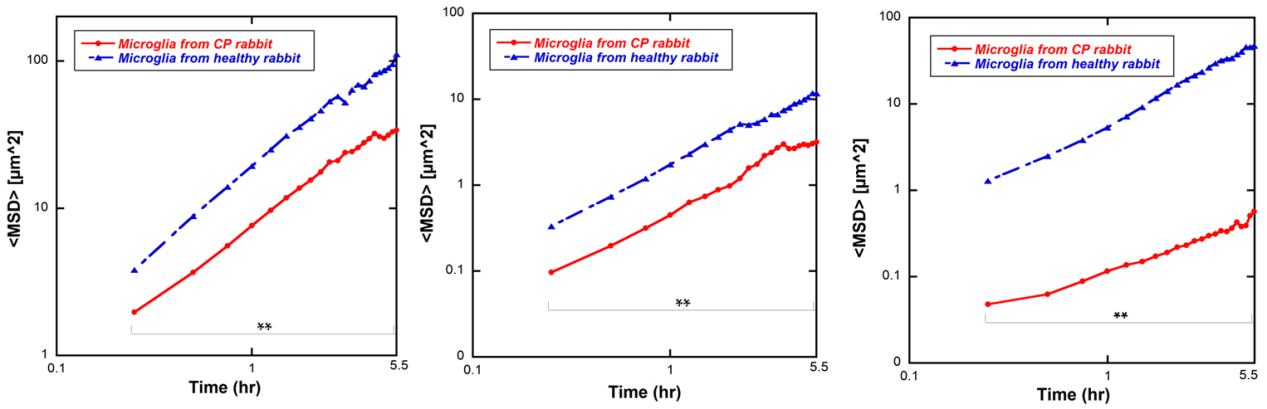


Comparison of the mean square displacement <MSD> of microglial cells from CP (Red) and Healthy (blue) slices during live-imaging in 3 different brain slices. The <MSD> was plotted in a log scale as a function of time. Mixed model ANOVA analysis showed that in each group, the <MSD> of microglia from CP is different from that of healthy brain. **p<0.01.

**Figure S3 –** **Confocal image of dendrimer treated brain slices (healthy, taken under 40X magnification) at 2 different time points after D-Cy5 treatment (1h, 4h). Blue: DAPI, Green: Anti-Iba1 (antibody labelling microglia), Red: D-Cy5. Scale bar: 50 μm.**


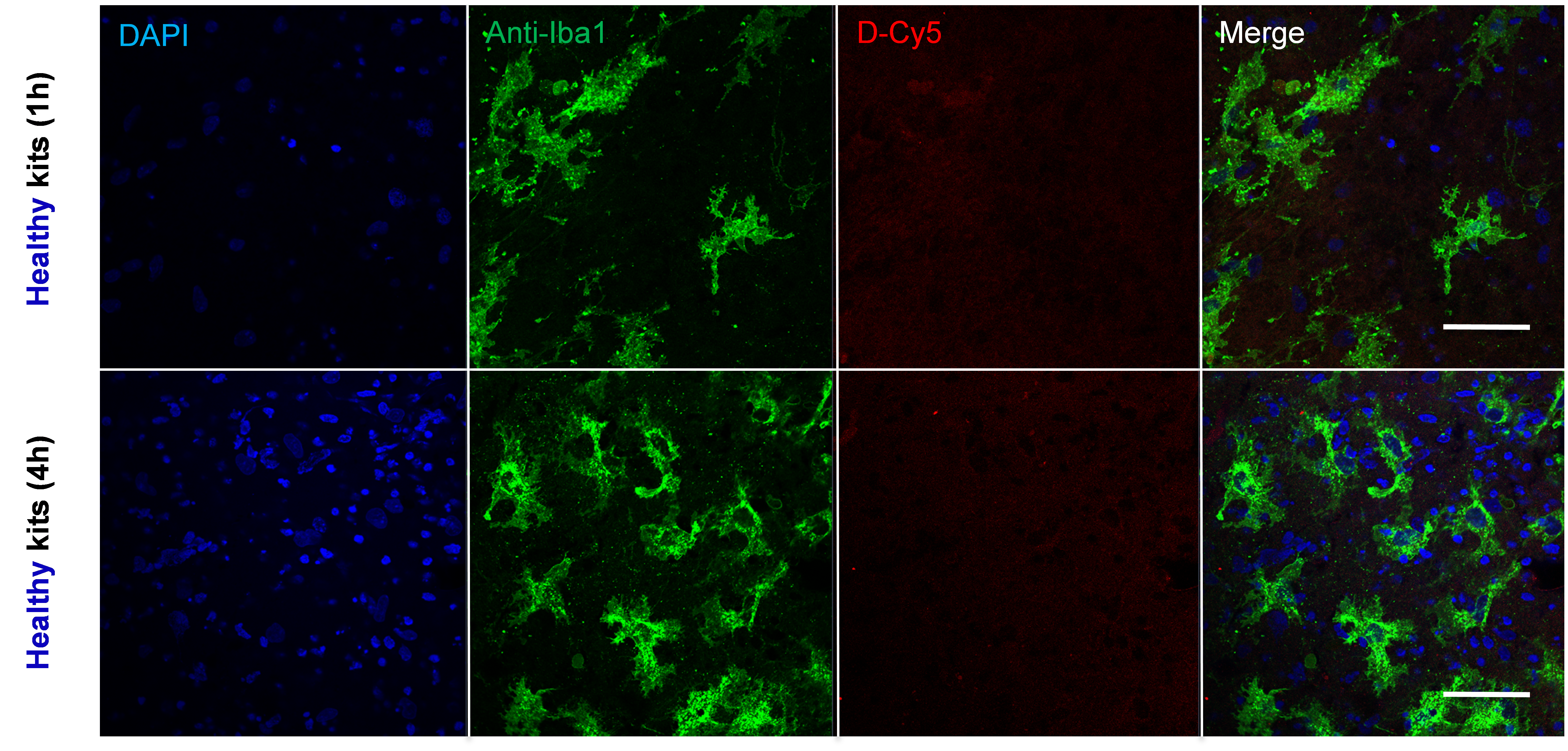


**Table S1 – One-way ANOVA analysis for Figure 1: the LDH release by neonatal rabbit brain slices as an indicator of tissue viability during incubation. * p<0.05, ** p<0.01, *** p<0.001, **** p<0.0001.**

| Days after incubation | 0.125 | 0.25 | 0.79 | 1.125 | 3 | 5 | 6 | 7 | 10 |
| --- | --- | --- | --- | --- | --- | --- | --- | --- | --- |
| 10 | **** | **** | **** | **** | **** | **** | * |  | N/A |
| 7 | **** | *** | **** | ** | ** | * |  | N/A |  |
| 6 | **** |  | * |  |  |  | N/A |  | * |
| 5 | *** |  |  |  |  | N/A |  | * | *** |
| 3 | ** |  |  |  | N/A |  |  | ** | **** |
| 1.125 | ** |  |  | N/A |  |  |  | ** | **** |
| 0.79 |  |  | N/A |  |  |  | * | **** | **** |
| 0.25 | * | N/A |  |  |  |  |  | *** | **** |
| 0.125 | N/A | * |  | ** | ** | *** | **** | **** | **** |

LDH level was significantly lower at 0.125 days, which is probably indicative of the initial cell death with sectioning the slices. The LDH levels then stabilized between 0.25 days and 5 days with no significant difference between the groups indicating no increase in cell death. LDH levels increased from day 6 to day 10 indicating increased cell death and decreased tissue viability at later time points. All experimental procedures were done between 0.25-1.125 days which was well within the time point of maximal tissue viability.
